# Peptidome Profiling of *Bubalus bubalis* Urine and Assessment of Its Antimicrobial Activity against Mastitis-Causing Pathogens

**DOI:** 10.3390/antibiotics13040299

**Published:** 2024-03-26

**Authors:** Rohit Kumar, Nikunj Tyagi, Anju Nagpal, Jai Kumar Kaushik, Ashok Kumar Mohanty, Sudarshan Kumar

**Affiliations:** 1 Cell Biology and Proteomics Lab., Animal Biotechnology Centre, ICAR-National Dairy Research Institute, Karnal 132001, Haryana, India; 2 ICAR-Indian Veterinary Research Institute, Mukteshwar 263138, Uttarakhand, India

**Keywords:** antimicrobial peptide, urinary peptides, bioactive peptides, proteases, buffalo, mass spectrometry

## Abstract

Urinary proteins have been studied quite exhaustively in the past, however, the small sized peptides have remained neglected for a long time in dairy cattle. These peptides are the products of systemic protein turnover, which are excreted out of the body and hence can serve as an important biomarker for various pathophysiologies. These peptides in other species of bovine have been reported to possess several bioactive properties. To investigate the urinary peptides in buffalo and simultaneously their bioactivities, we generated a peptidome profile from the urine of Murrah Buffaloes (n = 10). Urine samples were processed using <10 kDa MWCO filter and filtrate obtained was used for peptide extraction using Solid Phase Extraction (SPE). The nLC-MS/MS of the aqueous phase from ten animals resulted in the identification of 8165 peptides originating from 6041 parent proteins. We further analyzed these peptide sequences to identify bioactive peptides and classify them into anti-cancerous, anti-hypertensive, anti-microbial, and anti-inflammatory groups with a special emphasis on antimicrobial properties. With this in mind, we simultaneously conducted experiments to evaluate the antimicrobial properties of urinary aqueous extract on three pathogenic bacterial strains: *S. aureus*, *E. coli*, and *S. agalactiae*. The urinary peptides observed in the study are the result of the activity of possibly 76 proteases. The GO of these proteases showed the significant enrichment of the antibacterial peptide production. The total urinary peptide showed antimicrobial activity against the aforementioned pathogenic bacterial strains with no significant inhibitory effects against a buffalo mammary epithelial cell line. Just like our previous study in cows, the present study suggests the prime role of the antimicrobial peptides in the maintenance of the sterility of the urinary tract in buffalo by virtue of their amino acid composition.

## 1. Introduction

The urinary tract is under the constant stress of managing and expelling the waste product, and urinary tract infection from invading pathogens doubles this stress. To counter the effect of invading pathogens, innate immunity comes into play with its best tool—Anti Microbial Peptides (AMPs). Urine harbors thousands of proteins and peptide sequences. Peptides originate from proteins as a result of proteolytical cleavages of the parent proteins by endogenous proteases [1,2,3,4,5]. The physiology and pathophysiology of animals are dynamic, and urine encapsulates these ever-changing events in the body better than any other biological fluids. Urine is a relatively more stable biological fluid for peptidomics, as the process of proteolytic cleavage of proteins is completed by the time of urine voiding [6]. All these attributes make urine an excellent sample for analysis, profiling, and biomarker discovery. The urine peptidome has been immensely explored in the search for a biomarker associated with several pathophysiologies, such as urothelial bladder cancer, chronic kidney disease, diabetic kidney disease, and rheumatoid arthritis [7,8,9].

Various reports suggest that Antimicrobial Peptides (AMPs) play a pivotal role in maintaining a sterile environment in the urinary tract [10,11,12,13,14]. AMPs work as the first line of innate immunity and neutralize bacterial invasion by disrupting membranes, ion sequestration vital for bacterial growth, and inhibiting replication and translation machinery [15]. AMPs regulate the immune system and modulate its inflammatory mechanisms in order to serve a protective role for host organisms against harmful microbes, including bacteria, fungi, and viruses [16], by acting as a chemotactic factor and assisting in recruitment of immune cells at the inflammation site [17]. Additionally, the AMPs participate in several vital processes such as angiogenesis, wound healing, phagocytosis, apoptosis, and cytokine release [18,19,20,21,22,23]. Several strategies have been adopted to increase the efficiency and bioavailability of the AMPs. Delivery systems such as lipid carriers, polymer carriers, silica-based nanoparticles, and metal nanosystems are being used for efficient delivery of AMPs [24]. Heterologous expression systems of peptides have emerged recently and are gaining traction because they offer several advantages by harnessing fusion tags, tandem multimeric expression, promoters, and strains appropriate to the specific characteristics of each AMP [25,26]. Moreover, recent advancement in machine learning pipelines and deep generative models has accelerated the identification of potential AMPs [27]. Considering their multifaceted role, AMPs can be a way forward to counter the growing incidences of multi drug resistance.

Excessive use of traditional antibiotics in the treatment of mastitis in the commercial dairy sector has resulted in the development of multi-drug strains which not only threaten the well-being of livestock’s life but also humans. The annual economic loss sustained by the global dairy industry on account of udder infections has been projected to be EUR 16–26 billion [28]. The annual economic loss due to bovine mastitis is estimated to be 7165.51 crores in India, of which, 57.93% (4151.16 crores) has been attributed to sub-clinical mastitis [29]. A survey conducted by the Centre for Disease Dynamics Economics & Policy in India recommended phasing out the sub-therapeutic use of antibiotics in animals and changing incentives to discourage the use of antibiotics. We are investigating endogenous peptides/AMPs as a possible alternative to antibiotics. In Indian tropical climatic conditions, *S. aureus, E. coli*, and *S. agalactiae* were prevalent mastitis-causing pathogenic bacterial strains in dairy herds [30]. Hence, we especially targeted these strains in our study.

Profiling of urine endogenous peptides can open a new dimension for the discovery of peptide-based drugs. While profiling can be comparatively easier, the tedious job of selection, evaluation, and scrutiny of hundreds of peptides for antimicrobial activity can be a hurdle. To overcome these hurdles, various web-based prediction platforms can be used to make the selection process convenient. The present study is second in line with our previous study to obtain a deeper insight into the functionality of urinary peptides in dairy animals. This study side by side compares the nature, physicochemical characteristics, and origin of peptides from our previous study and discusses the functional aspect of the generated peptides and how their compositional biases affect their functionality. Additionally, the study also shows that urinary peptides exert functional antimicrobial activity and can be further investigated to explore their applicability in the dairy industry.

## 2. Results

### 2.1. Molecular Nature of Buffalo Urinary Peptides

**Figure 1:**
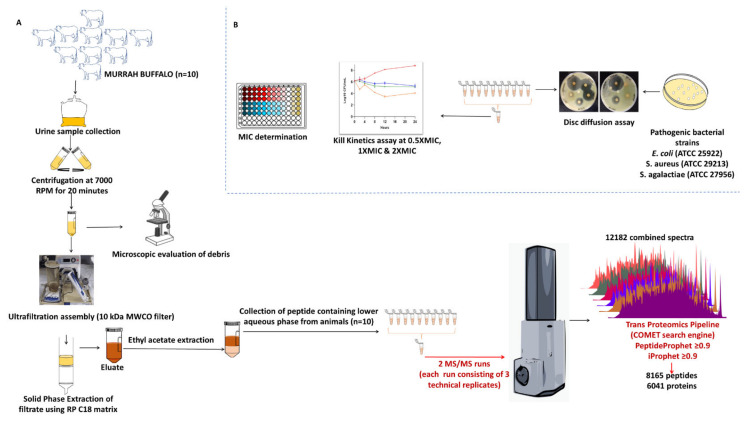
Sample processing, peptide extraction, and confirmation of activity: (**A**) Urine sample collection, processing, extraction of the peptide from the urine and its analysis using nLC-MS/MS. (**B**) Functional validation of antimicrobial activity of urinary peptide extract.

**Figure 2:**
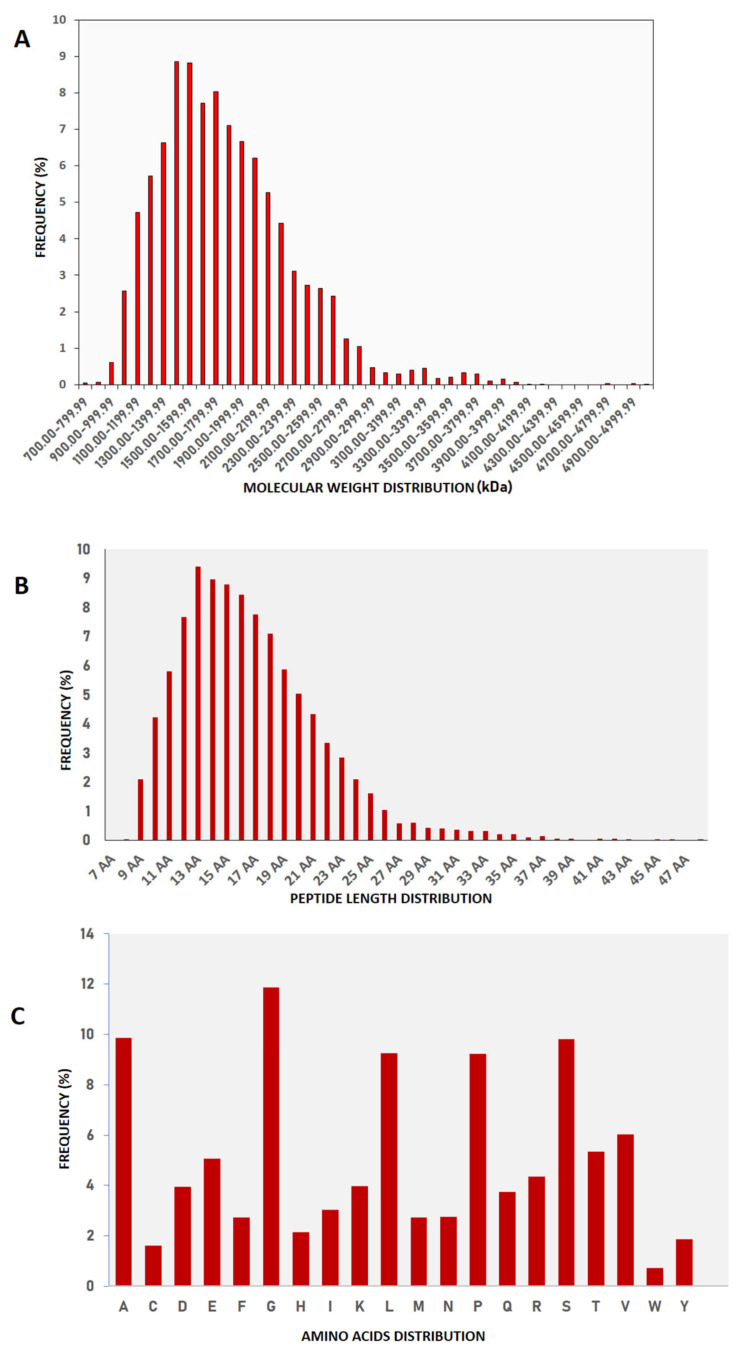
Frequency distribution: (**A**) Molecular weight, (**B**) Peptide length, and (**C**) Compositional amino acids of buffalo urinary peptides.

**Figure 3:**
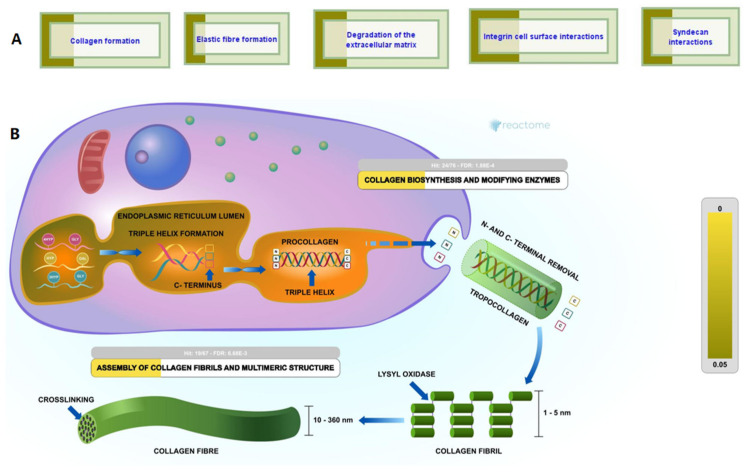
Origin of urinary peptides: (**A**) Maximum hits obtained in the ECM organization pathway of Reactome, (**B**) Urinary peptides are ECM derived and involved in biosynthesis of collagen.

We identified a total of 8165 peptide sequences in the buffalo urinary aqueous extract originating from 6041 proteins of systemic and local origin (Supplementary Table S1) using ultrafiltration, solid phase extraction and mass spectrometry (Figure 1). The frequency distributions of the retrieved sequences were created to find the pattern in molecular weight, peptide length, and amino acid composition. Our study was focused on small molecular weight endogenous peptides from urine; by using a 10 kDa molecular weight cut-off membrane, larger-sized peptides and proteins were separated from the smaller peptides. We observed similar trends in our results; the molecular weight of retrieved peptide sequences was in the range of 700 Da–5000 Da (Figure 2A,B). Sequences with a molecular weight of 1.4–1.5 kDa were relatively abundant. Tricine gel visualization also indicated the presence of <10 kDa peptides in the urine aqueous extract. To understand the pattern observed in the molecular characteristics of urinary peptides, proteases were investigated that might have a possible role in the release of sequences from the parent protein (discussed in later sections). The average length of peptide sequences was 16 amino acids, with peptides of 13 amino acids length being the most prevalent. In terms of amino acid composition, we identified alanine, glycine lysine, leucine, and serine as the abundant amino acid residues in buffalo urinary peptides (Figure 2C). Most of the peptide sequences originated from the extracellular matrix (ECM), particularly collagen and integrin isoforms (Figure 3). Collagen biosynthesis and assembly of collagen fibrils and multimeric structure pathways gave maximum hits in the ECM organization pathway, with FDR 1.88 × 10^−4^ and 6.68 × 10^−3^, respectively (Figure 3). The results suggested that the peptide sequences are highly variable, but their molecular attributes tend to follow a certain pattern.

### 2.2. Proteases Involved in Urinary Peptide Generation

**Figure 4:**
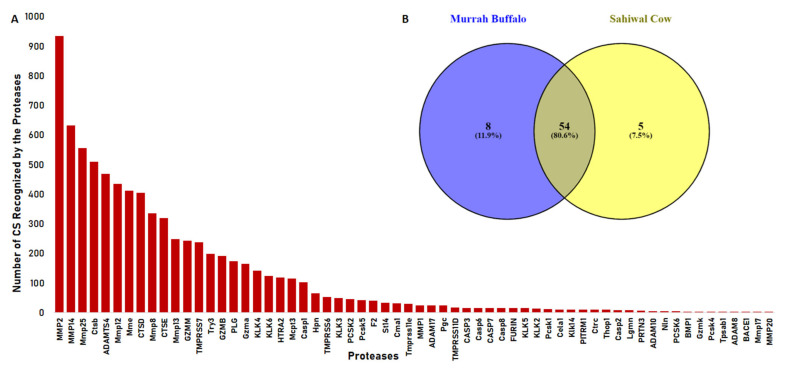
Proteases involved in proteolytic events: (**A**) Frequency distribution of cleavage sequences recognized by the different proteases. (**B**) Common proteases identified in Murrah buffalo and Sahiwal cow (Reported in our previous work).

**Figure 5:**
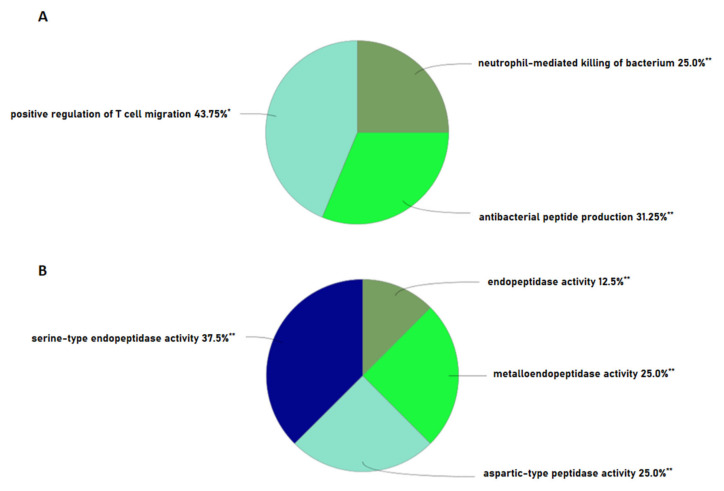
Gene Ontology: Enrichment of (**A**) immune system functions of proteases, (**B**) molecular functions of proteases. Percentage enrichment values with (*) as a superscript are significant with *p* < 0.05 and values with (**) as a superscript are significant with *p* < 0.01.

**Figure 6:**
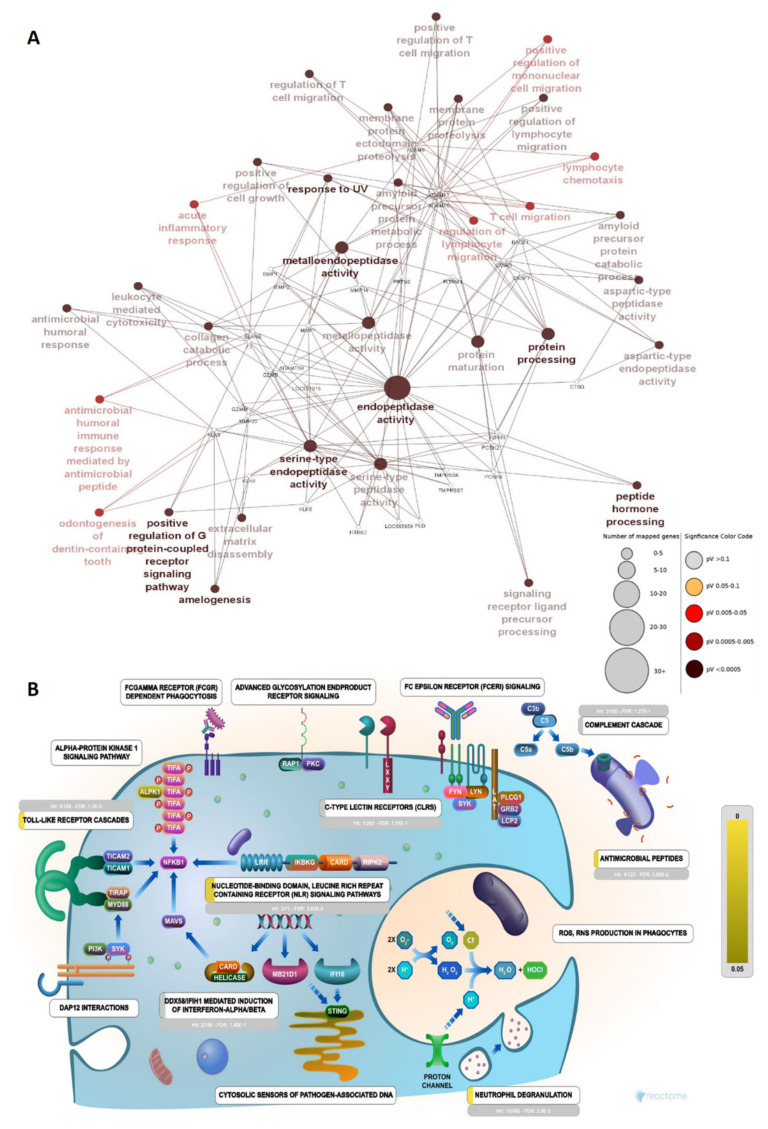
Biological process of proteases: (**A**) Network depicting proteases (small, white-colored nodes) and corresponding molecular function or activity. The size of the nodes corresponds to the number of mapped genes and the color denotes the significance of the GO term. (**B**) The number of genes mapped to different functions of the innate immune system in Reactome; four genes were mapped significantly to antimicrobial peptides, with 3.85 × 10^−2^ FDR.

To shed some light on the proteases that might be involved in peptide cleavage from the parent protein, the Proteasix tool was used for the prediction of involved proteases from the batch of peptide sequences. It uses N and C terminal information from peptide sequences and cleavage site information from the MEROPS peptidase database to predict the protease responsible for the release of resultant peptides. Proteases such as MMP2, MMP14, Mmp 25, Ctsb, ADAMST4, Mmp 12, Mme, and CTSD were actively involved in the cleavage and recognized most cleavage sites in the prediction (Figure 4A) (Supplementary Table S4). We also analysed our data with the observed cleavage function in Proteasix and found CTSS, CTSK, CASP6, MEP1B, CTSL, and MMP7, exclusively. The remaining seven proteases (TMPRSS6, Try3, ELANE, CTSG, MMP2, GZMM, and PLG) were common to both predicted and observed cleavages. Earlier, we performed a similar study in bovine urinary peptidome analysis. In comparison, we found more than 80.6% similarity between the proteases of Bos indicus and *Bubalus bubalis* (Figure 4B). Next, we performed the gene ontology of the proteases to understand their functions in various pathways. The predicted proteases bear metalloendopeptidase, serine-type endopeptidase activity, and aspartic-type peptidase activity (Figure 5B). The network showed the significant enrichment of endopeptidase, metalloendopeptidase, serine-type endopeptidase activity, and protein processing, with a relatively large number of genes mapped to these functions (Figure 6A). Reactome pathway analysis showed significant hits in functions viz. antimicrobial peptides, toll-like receptor cascades, neutrophil degranulation, nucleotide-binding domain, and leucine-rich repeats containing receptor signaling pathway (Figure 6B). Immune function ontology showed enrichment of positive regulation of T cell migration, antibacterial peptide production, and neutrophil-mediated killing of bacteria (Figure 5A). 

### 2.3. Bioactivities and Compositional Biases of the Predicted Functions

**Figure 7:**
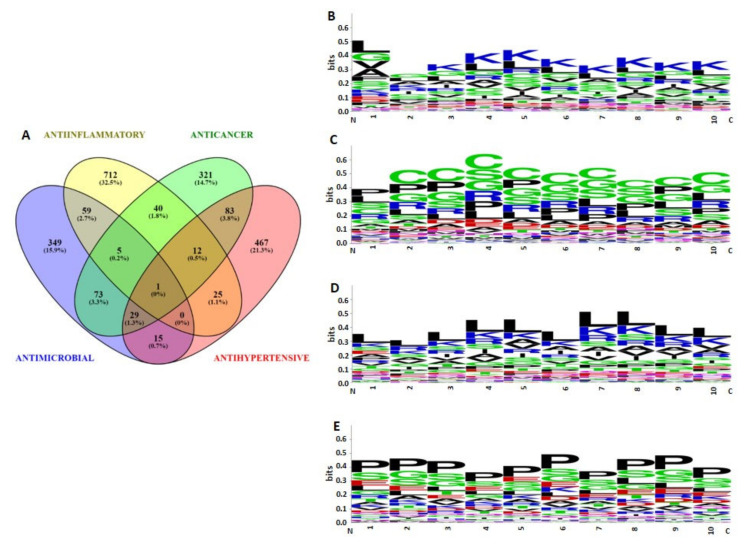
Predicted bioactivities of peptides: (**A**) Venn diagram showing unique and shared peptide sequences by different predicted bioactivities. N terminal (10 amino acids) sequence logos: (**B**) Antimicrobial peptides, (**C**) Anticancer sequences, (**D**) Anti-inflammatory sequences, (**E**) Antihypertensive sequences.

Peptides were classified into four groups viz. anti-microbial, anticancer, antihypertensive, and anti-inflammatory, based on their Support Vector Machine algorithm scores (≤0.9). The number of bioactive sequences predicted were as follows: antimicrobial (n = 531), anticancer (n = 564), anti-inflammatory (n = 854), and antihypertensive (n = 632) (Supplementary Table S3). A Venn diagram showed that antimicrobial and anticancer sets shared a relatively large number of sequences among them (n = 79), followed by anti-inflammatory and antimicrobial sets, sharing 59 sequences. A total of 29 sequences possessed anticancer, antimicrobial, and antihypertensive activity. However, only one sequence was predicted to have all four types of bioactivities (Figure 7A). 

N-termini of sequences were analyzed, in which the first ten amino acid residues were analyzed to determine the compositional biases of the sequences possessing different bioactivities. Antihypertensive sequences showed proline as the dominant residue at each position, followed by serine and glycine (Figure 7E). Meanwhile, anti-inflammatory sequences were predominated by leucine at each position, followed by lysine and arginine (Figure 7D). This trend was reversed in antimicrobial sequences, lysine was dominant at almost every position, followed by leucine (Figure 7B). However, the first residue from the N-terminus was predominated by leucine and glycine amino acids. Interestingly, more than one amino acid showed prevalence in anticancer sequences, almost every position was predominated by cysteine, followed by serine, glycine, proline, and arginine residues (Figure 7C).

### 2.4. Antimicrobial Assay

**Figure 8:**
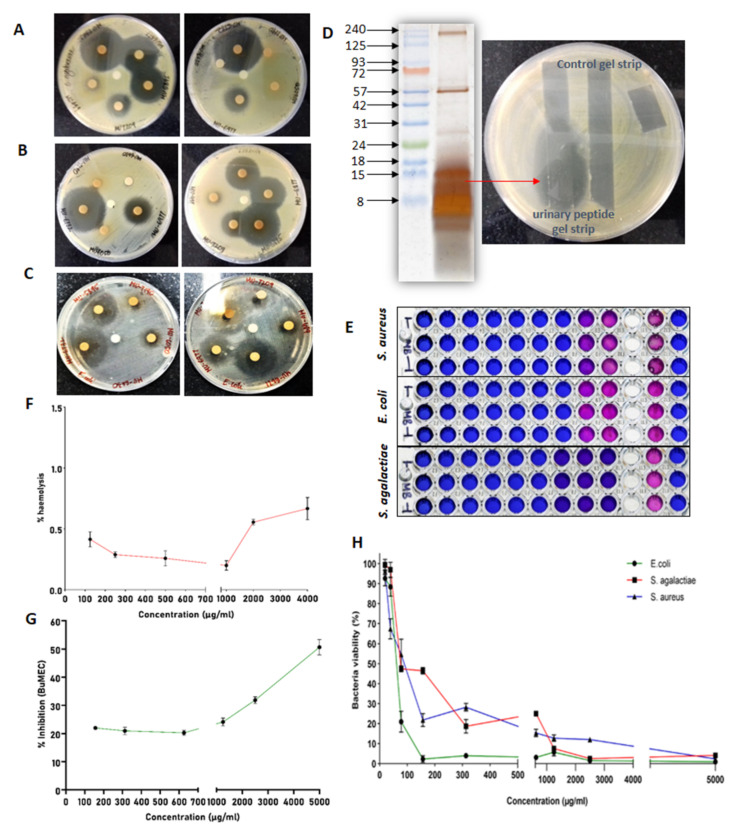
Antimicrobial and cytotoxicity assays: Disc diffusion assay of urinary peptide extract against (**A**) *S. agalactiae*, (**B**) *S. aureus*, and (**C***) E. coli*. (**D**) Visualization of the extracted urinary peptide using tricine SDS-PAGE and confirmation of antimicrobial activity of urinary peptide by overlaying the unstained gel strip over inoculated agar surface. (**E**) Broth microdilution assay in conjunction with resazurin dye. (**F**) Percent hemolysis exerted by urinary peptide on the RBCs. (**G**) Cytotoxicity exerted by urinary peptide on BuMEC cell line. (**H**) Determination of bacterial viability in the concentration-dependent manner of urinary peptide extract.

A zone of inhibition was visualized after the overnight incubation for each animal to confirm the antimicrobial activity of urinary peptide extracts. The urinary peptide extracts from some animals’ urine significantly inhibited the growth of bacteria and showed the development of a larger zone compared to other urine samples. After confirmation of the antimicrobial activity in buffalo urinary peptides, urinary peptide extracts from different samples were pooled and used for the determination of MIC and kill kinetics of the extract against *S. aureus*, *E. coli*, and *S. agalactiae* (Figure 8A–C). We further associated the antimicrobial activity to urinary peptides by overlaying an unstained tricine-SDS gel strip over an inoculated agar. The urinary peptide showed the zone of inhibition which can now be directly associated with the urinary peptides present in the aqueous extract of C18 eluate (Figure 8D).

MIC determination was performed by broth microdilution assay in conjunction with resazurin dye to spot the wells with viable and non-viable bacteria. The MIC of buffalo urinary aqueous extract against *S. aureus*, *E. coli*, and *S. agalactiae* was 78.125 mg/L, 78.125 mg/L, and 156.25 mg/L, respectively. The MIC was determined by visualizing the color of resazurin dye 2 h post incubation (Figure 8E). Wells with blue colored resazurin show that bacteria were either dead or were fewer in number, whereas pink or purple colored dye showed the presence of a viable population of bacteria. 

Except *S. agalactiae*, the bacterial viability for *S. aureus* and *E. coli* sharply declines above 80 mg/L concentration. No bacterial strains were viable at 5 g/L concentration, which was the highest concentration used in MIC determination (Figure 8F).

### 2.5. Kill Kinetics Assay

**Figure 9:**
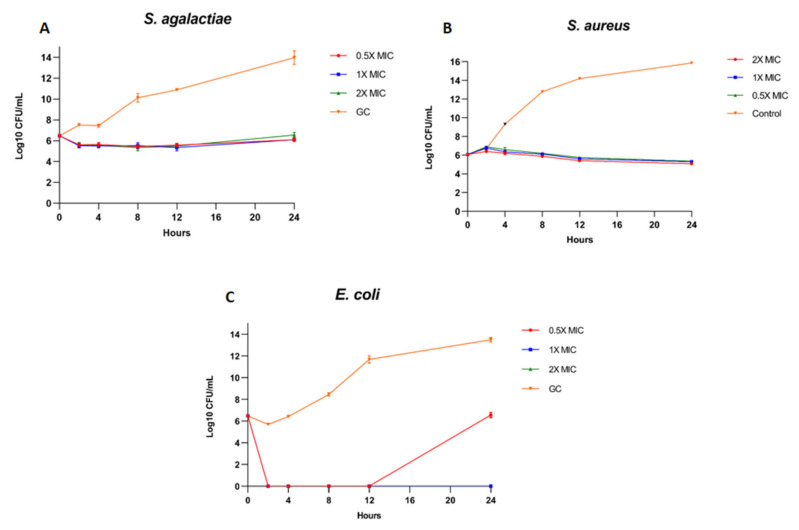
Kill kinetics curve of urinary peptide extract against: (**A**) *S. agalactiae*, (**B**) *S. aureus*, and (**C**) *E. coli*.

1 × MIC, 2 × MIC of the buffalo urinary peptide extract completely inhibited the growth of *E. coli,* with more than a 3Log reduction in bacterial CFU, suggesting that these two concentrations had a bactericidal effect on *E. coli* (Figure 9C). However, bacterial growth resumed after 12 h at 0.5 × MIC concentration. In the case of *S. agalactiae* and *S. aureus*, all three concentrations of urinary peptide extract maintained the bacterial CFU to the level of initial inoculum. The buffalo urinary peptides had a bacteriostatic effect against *S. aureus* and *S. agalactiae,* as the CFU counts remained equivalent to the initial CFU even after 24 h of incubation. The results suggest that the buffalo urinary peptides are more active against Gram negative bacteria than Gram positive. Theoretically, a 2× concentration of MIC would have shown significant inhibition compared to 1×, as evident from the broth microdilution assay. However, the initial inoculum for the kill kinetics assay had more than 5 log bacteria, whereas for the broth microdilution assay, it was only 5 log.

### 2.6. Haemolysis and Cytotoxicity Assay

Buffalo urinary aqueous extract showed negligible hemolysis of RBCs even at higher concentrations, while it inhibited around 40% of the BuMEC cell population at 5 mg/mL concentration (Figure 8G,H).

## 3. Discussion

Peptides obtain access into the urine once their circulating concentration exceeds the tubular saturation limit, explaining the origin of various systemic proteins in the urine in our data [31]. By scanning MS/MS spectra ranging from 200 to 2200 *m*/*z*, we identified a total of 8165 peptides, with a molecular weight ranging from 700–5000 Da. We obtained a similar pattern of molecular weight distributions in our previous findings [32]. The predominating amino acid residues in the sequences were Ala, Gly, Leu, Pro, and Ser. We found similar predominating amino acid residues in cow urine, except for Lys, Arg, and Val. Most of the peptide sequences originated from the extracellular matrix, especially collagen and integrin. Previous studies have established collagen-derived peptides as abundant peptides in urine [9,33,34]. We obtained maximum hits in the ECM organization pathway in Reactome and found the enrichment of collagen biosynthesis and assembly of collagen fibrils and multimer pathways. Collagen has been shown as a key player in bladder compliance, proliferation, and bladder filling [35,36]. Collagen, being an important constituent of ECM, is targeted by the endogenous proteases during ECM degradation, and hence shows its manifestation in urine in the form of small peptides. As collagen peptides escape tubular reabsorption, the relative abundance of collagen-derived peptides in plasma and urine showed a significant correlation [34]. 

Proteases do not work individually but rather in cascades. When more of such cascades come together with different classes and families of proteases, a more complex Protease web is formed. Interference in this web can cause diseases such as cancer, thrombosis, atherosclerosis, cystic fibrosis, Chron’s disease, chronic kidney disease, and neurodegenerative disease [37,38,39]. The heightened or lowered activity of certain proteases can be associated with a pathophysiological condition. A multidimensional approach is necessary to understand the outcome of changes in protease activity due to its complex regulatory mechanisms. Owing to certain conditions, changes in the levels of proteases, activation, and inhibition can result in different outcomes in proteolytical events, which ultimately decide the peptidome. We predicted a total of 76 proteases from the Proteasix-based prediction, out of which 66 with ≥70% specificity were reported in final data. Further, the ontological analysis of the proteases provided insights into the classes and their participation in certain events. Referring to our investigation in cows, we found more than 80.6% similarity between the proteases of buffaloes and cows (Figure 4B) [32]. Immune functions’ ontology showed the enrichment of antibacterial peptide production (31.25%), a function relevant in our search of antimicrobial peptides in urinary peptidome. A similar study in humans showed the enrichment of antimicrobial peptide production in immune function [40]. A large number of proteases were mapped to endopeptidase and serine-type endopeptidase activity function with high significance. We obtained serine type, aspartic type, and metalloendopeptidase activity as the top enriched functions in molecular function ontology. We found a significant percentage of MMPs in our data; these proteases are involved in the degradation of ECM proteins and are involved in diverse biological events [41].

The frequent occurrence of certain amino acids can be associated with distinct bioactivity, whereas particular residues showed their signature in all bioactivities. Gly residue was a frequently occurring amino acid in all types of bioactivities. The Gly residue constituted 10.98%, 10.88%, 10.79%, 10.77%, and 11.82% of the antibacterial peptides (ABP), anticancer peptides (ACP), antifungal peptides (AFP), antiparasitic peptides (APP), and antiviral peptides (AVP) [42]. Similarly, the Leu residue accounted for 10.88% of ABPs, ACPs, AFPs, APPs, and AVPs [43].

AIPs preferred Leu, Lys, and Arg residues in abundance in their sequences [44]. One study reported Leu residue to be a commonly occurring residue in AIPs [45]. The presence of a Leu, Lys rich peptide has a key role in the induction of anti-inflammatory cytokines. Similarly, our results presented Leu, Lys, and Arg as abundant residues in anti-inflammatory sequences, which substantiates the previous findings. Compositional analysis of the AMPs versus non AMPs dataset showed that the average composition of Lys, His, Ala, Iso, Leu, Pro, and Try in AMPs were relatively higher compared to non-AMPs [46]. Several studies have pointed out the critical role of Lys residue in AMPs. Antimicrobial peptides against Gram + ve and Gram − ve bacteria showed 9.26% and 7.47% Lys residue composition. The abundance of Leu, Gly, and Lys were higher in AMPs, whereas Arg residue showed relatively higher abundance in mammalian-derived AMPs [47]. We observed a similar trend in our study, Lys residue was predominantly present at almost every position, followed by Leu and Gly residues. The cores of AMPs are mostly abundant in Gly and Lys residues [48]. Meanwhile, Lys residue is a predominant residue at N-terminals, preferentially present at 7, 8, 11, 12, 14, and 15 positions [46]. In line with this finding, our finding showed that Lys residue occupied 3–10 positions in AMP sequences. The average hydrophobicity and charge of antimicrobial sequences were −0.21 and +2.85, respectively. We employed the web-based server for the prediction of bioactivities. However, certain residues are more preferred for a particular bioactivity, e.g., AMPs are mostly rich in Lys and Arg residues. The predicted antimicrobial sequences in our data were preferentially rich in Lys residue. The presence of cationic residues can improve the efficacy of peptides against the negatively charged bacterial membrane [49]. Research has also reported that the combination of cationic AMPs can increase the transmembrane potential, which favors the permeabilization of the bacterial membrane [50]. Antimicrobial sequences have been shown to exert anti-inflammatory activity [51]. Our data showed that 59 (2.7%) sequences are common among antimicrobials and anti-inflammatories sets. AMP sequences exert this activity by binding to LPS and simultaneously inhibiting the release of LPS-induced pro-inflammatory NO [52].

Similar to AMPs, ACP sequences were also predominated by Gly, Lys, and Leu residues. The presence of residues such as Arg and Lys in AMP and ACP sequences are responsible for the disruption of the cell membrane [53]. ACP amino acids’ distribution was skewed towards basic Arg and Lys residues (26%). Meanwhile, hydrophobic Leu and Ile constituted 19% of the ACP sequences [54]. Like AMPs, both positive net charge and hydrophobicity are needed for the functioning of ACPs; however, the anticancer activity does not improve by increasing the net charge and hydrophobicity beyond a certain limit. A reciprocity between these two factors is important for the proper functioning of ACPs [55]. Our finding showed Cys, Gly, and Pro as frequently occurring amino acids. The amino acid distribution pattern was aberrant considering the reports made by previous studies, but a recent study backed Gly and Cys as abundant amino acids in ACPs [56]. 

In line with our previous findings, we observed a similar pattern of amino acid distribution in all predicted bioactivities except anticancer sequences. In the case of the buffalo anticancer sequence, Cys, Gly and Pro were predominating residues, whereas in cow anticancer sequences, only Gly and Pro were abundantly distributed throughout the sequence length, while the Cys residue was completely absent [32]. 

To confirm the antimicrobial nature of the total urinary peptides, we performed a disc diffusion assay for every individual sample. The activities of some samples were relatively significant, as confirmed by the size of the zone of inhibition. The antimicrobial activity can be associated with the urinary peptides present in the C18 eluate of the Murrah buffalo urine. However, this antimicrobial activity can be affected temporally with changing environments and possibly by the diet of the animal. These factors were not taken into consideration in our study and must be investigated to from a firm conclusion about the antimicrobial nature of urinary peptides. The peculiarity in our findings was the large differences in the antimicrobial activity. Some urinary peptide samples showed strong antimicrobial activity with the development of a large zone of inhibition. Meanwhile, in some cases, activity was either feeble or totally absent. To explain the variable activity of extracted urinary peptides from different animals, biological replicates must be explored in a mass spectrometry-based approach to find the peptides for the underlying activity and associated quantities in different samples. In our study, the MIC of urinary aqueous extract was higher against *S. agalactiae* relative to *S. aureus* and *E. coli*. The MIC of buffalo urinary aqueous extract against *S. aureus, E. coli*, and *S. agalactiae* were as follows: 78.125 mg/L, 78.125 mg/L, and 156.25 mg/L. Furthermore, the kill kinetics studies revealed that urinary peptide extract completely inhibited the growth of *E. coli* at 1 × MIC and 2 × MIC concentrations. Meanwhile, urinary peptides showed bacteriostatic activity against *S. aureus* and *S. agalactiae* at 0.5×, 1×, and 2 × MIC concentration. Besides our previous study in Sahiwal cows, we found only one report supporting the antimicrobial nature of the total urinary peptides. The study reported the antimicrobial activity of goat urinary peptides against *S. aureus* and *E. coli*, with MIC of 0.0199 µg/µL and 0.039 µg/µL, respectively [57]. However, the study was focused on the activity of cationic antimicrobial peptides, which were isolated from the goat urine using weak cation exchange beads. On the contrary, we isolated the total urinary peptides using SPE irrespective of their nature (anionic or cationic) to capture as much of sequence as possible. Furthermore, our hemolysis and cytotoxicity experiment data suggested that buffalo urinary aqueous extract was safe on RBCs and the BuMEC cell line. Due to the tedious sample processing, it was difficult to extract information from the larger sample size. However, a larger sample size can provide information about the existing variations in antimicrobial activity in urinary peptides. However, to exactly identify the reason behind the variations will be a challenging task. Our study shows that urinary peptides, like many other components (metabolites), are responsible for antimicrobial activity. These peptides are responsible for maintaining the sterile environment inside the urinary tract, but dilution might downplay their overall antimicrobial activity. The significant antimicrobial activity we obtained in our study is due to the enrichment of the urinary peptides from the larger volumes of urine samples.

## 4. Materials and Methods

### 4.1. Sample Collection and Processing

The urine samples were collected from ten healthy female Murrah buffaloes (*Bubalus bubalis*). The sample for MS analysis was created by pooling and was processed separately with three technical replicates. Fixed-time morning void urine samples (approx. 500 mL) were collected by massaging the perineum of the animal. The samples were transferred to the lab and filtered through a muslin cloth to remove any contaminating particulate matter, followed by centrifugation at 7000 rpm for 20 min to allow settlement of any cell debris or particulate matter. The microscopic examination was performed for individual samples, before and after the centrifugation, to observe the presence of RBCs, WBCs, other cells, and debris. The purified clean urine was further used for peptide extraction and purification (Figure 1).

### 4.2. Peptide Extraction

The supernatant obtained after centrifuging urine samples was filtered through ultrafiltration assembly (Thermo easyload Masterflex, model 7518-00, Brrington, IL, USA) with a 10 kDa molecular weight cut-off filter (Pall MinimateTM TFF Capsule) to separate the small molecular weight endogenous peptides from the high molecular weight proteins. The filtrate obtained from ultrafiltration assembly was used for Solid Phase Extraction (SPE) (Figure 1). For SPE, the pH of the obtained filtrate was adjusted to ≤3, using trifluoroacetic acid (TFA). The column was prepared by packing the swollen C-18 reversed-phase silica. Briefly, 20 g of the matrix was resuspended in methanol, the slurry was prepared and packed into a column by continuously stirring the matrix in methanol and slowly pouring it into the column, followed by several washes with methanol. Post packing, the column was conditioned and equilibrated by 90% methanol and 10 column volumes of 0.1% TFA. The packed column was conditioned using 90% methanol followed by equilibration with 10 column volumes of 90% methanol containing 0.1% TFA. After equilibration, PM was loaded with a flow rate of 0.5 mL/min, followed by desalting using 5% methanol with 0.1% TFA. The desalted peptides were eluted by 60% acetonitrile (ACN) with 0.1% TFA. Processing of around 500 mL of urine yielded approx. 30–50 mL of eluate with dark brown appearance. For the removal of the dark brown substances (possible contaminating metabolites in urine) using ethyl acetate-based extraction was performed. The eluates were subjected to the double volume of ethyl acetate followed by end-to-end rotation for 5 min and were allowed to settle for 10 min to differentiate into two layers. The upper organic layer was stored appropriately for metabolome profiling and the lower aqueous phase was aliquoted in 2 mL microcentrifuge tubes and dried by speed vac (Thermo savant ISS110 SpeedVac concentrator, ISS110-230, Waltham, MA, USA). Extracted peptides were visualized by tricine SDS PAGE.

### 4.3. LC-MS/MS Acquisition

The peptides were initially enriched on a nano-trap column (C18, 2 cm, 5 µ, 100 Å, Agilent), followed by elution on to analytical column (ZORBAX 300SB-C18, 0.1 × 150 mm, 3.5 µ, Agilent). The peptides were sprayed using a nanoelectrospray emitter tip of 10 µm (Bruker, Germany), using 0.1% formic acid (FA) in water as solvent A and 0.1% FA in ACN as solvent B. The peptides were loaded onto the trap column using 97% solvent A, followed by resolution on the analytical column using a linear gradient of 2–45% solvent B for 55 min at a constant flow rate of 300 nL/min. The data were acquired in data-dependent acquisition mode, subjecting the six most intense ions in each survey scan to MS/MS analysis within an *m*/*z* range of 400–2200. The collision-induced dissociation (CID) method was used for precursor fragmentation, and the precursor ions selected for MS/MS fragmentation were excluded after every three spectra. The absolute threshold for precursor ions per 1000 summations was 1200 counts.

### 4.4. Data Processing

The raw data files were converted to mzML format using MSconvert GUI using the default parameters. The files were searched for MS/MS spectra against the UniProt Bos Taurus database using the Trans-Proteomic Pipeline version 5.1.0 released on 3 November 2017. For the analysis, the peptide assignments were performed using the Comet search engine. As peptides were undigested, the so cut everywhere option was selected for the search parameter. We selected N- and C-terminal unspecific digestion under enzyme search setting. Allowed missed cleavage was set to 2. The minimum peptide length parameter was set to 7 amino acid residues. Peptide Prophet and Protein Prophet were used to calculate the probability scores for both peptides and the corresponding proteins. A Peptide Prophet score of ≥0.99 was used for filtering out low scoring peptides and a final iProphet score of ≥0.99 was used for the protein identification. The final list of proteins was prepared with a ≥0.99 Protein Prophet score. 

### 4.5. Protease Prediction Using MEROPS Database

The Proteasix tool was employed to predict the protease responsible for the release of peptide sequence from the precursor protein. It uses N- and C-terminal information from the peptide entry list and MEROPS peptidase database for the prediction of the protease. To prepare the entry for Proteasix, the batch peptide match tool from Protein Information Resource (PIR) was used to retrieve the start and end amino acid position in a protein sequence. The list of proteases was sorted and the total number of cleavages for each protease was determined. The protease list obtained was used for gene ontology.

### 4.6. Bioactive Peptide Classification Using SVM Algorithm-Based Prediction Platforms

The bioactivities viz. antimicrobial, antihypertensive, anticancer, and anti-inflammatory for the peptide sequences were predicted using a high SVM score. A threshold value of 0.9 was set for the prediction, and sequences scoring less than 0.9 were culled from the study. Support Vector Machine (SVM) is an extensively used machine learning algorithm for designing the program for the prediction of bioactive peptides [58,59,60]. Anticancer activity was predicted using the tumorHPD server: http://crdd.osdd.net/raghava/tumorhpd/ (accessed on 2 October 2021). This tool utilizes 651 experimentally validated peptides (peptides binding to the tumor) in a positive dataset and 651 non-tumor binding peptides randomly generated from proteins obtained from SwissProt [59]. Antimicrobial peptides were predicted using the CAMPR3 server: http://www.camp3.bicnirrh.res.in/campHelp.php (accessed on 17 August 2021) [61]. The database has 10,247 antimicrobial peptides, 2915 antibacterial peptides, 1144 antifungal peptides, and 117 antiviral peptide entries. Antihypertensive sequences were retrieved from the AHTpin server: http://crdd.osdd.net/raghava/ahtpin/ (accessed on 27 August 2021). The database derives an ACE inhibitory peptides knowledgebase from AHTPDB, BIOPEP, ACEpepDB, and the literature, while negative datasets of uniform random fragments of proteins were acquired from Swiss-Prot [62]. Anti-inflam was accessed for the prediction of anti-inflammatory peptides: http://metagenomics.iiserb.ac.in/antiinflam (accessed on 10 October 2021).

### 4.7. Antimicrobial Assay and MIC Determination

The aqueous phase containing urinary peptides was resuspended in 100 µL Milli-Q water to assess the antimicrobial activity of the peptides by disc diffusion assay. The volume obtained after processing and C18 based solid-phase extraction constitute 4–6% (20–30 mL approx.) of total urine volume (400–500 mL). Post SPE, the urinary peptides were concentrated to 4–6% of total urine volume. One milliliter of the SPE eluate was aliquoted into 1.5 mL microcentrifuge tubes and lyophilized in speed vac. The lyophilized extract was reconstituted in 200 µL of Milli-Q water and 30 µL of the extract was coated onto 6 mm sterile discs (HIMEDIA, SD067-1VL). The urinary aqueous extract volume was concentrated almost 80 times before antimicrobial activity determination. The discs were allowed to dry under a laminar hood. A lawn of 0.5 McFarland equivalents of test cultures (*Staphylococcus aureus* ATCC 29213, *Escherichia coli* ATCC 25922, and *Streptococcus agalactiae* ATCC 27956) were swabbed on the surface of Mueller Hinton Agar (HIMEDIA, GM173-500G) and allowed to dry. With the help of sterile forceps, the coated discs were placed on the lawn of the test culture and incubated overnight. BSA digest was used as a negative control in the experiment. The appearance of the inhibition zone confirms the antimicrobial activity of the buffalo urinary peptides. Additionally, the tricine-SDS with Murrah buffalo urinary peptide gel strip was overlaid over the surface of agar inoculated with *S. aureus*. After overnight incubation, the antimicrobial activity was confirmed by the presence of a zone of inhibition.

MIC was determined using a Resazurin dye-based broth microdilution assay. The aqueous extract containing total urinary peptides obtained from buffalo urine was weighed and dissolved in Mueller Hinton Broth (MHB). One-hundred microliters of peptide solution was dispensed in each well of column 1 and 50 µL of MHB was dispensed in columns 2–9. Using a multichannel pipette, peptides from column 1 were double serially diluted in columns 2–9, resulting in 50 µL of peptides solution per well. The highest concentration used in in the assay was 5000 µg/mL and the lowest achieved through double serial dilution is 19.5 µg/mL. Column 11 containing 100 µL of standardized inoculums was taken as growth control for the experiment. Inoculums for the experiment were prepared by direct suspension of isolated colonies in normal saline from a 24-h agar plate and the turbidity of the suspension was adjusted to 0.5 McFarland indicator. The adjusted suspensions were then diluted by 1:20 in MHB, yielding approx. 5 × 10^5^ CFU/mL. Five microliters of the inoculums were dispensed in wells of column 1–9 and growth control column. The inoculums of the test cultures were prepared and dispensed within 15 min. The plate was sealed and incubated for 24 h at 37 °C. Using a multichannel pipette, 20 µL of the Resazurin dye was added to each well at 0.2 mg/mL concentration and then incubated for another 2–4 h. After incubation, the wells with slight color change were scored as MIC. To obtain a clear understanding of concentration-dependent changes in the bacterial survival rate, OD measurement was performed at 570 nm, with a reference wavelength of 600 nm and bacterial survival rate was calculated using the following formula:

% Bacterial viability = (OD_Treated_ − OD_negative control_)/(OD_positive control_ − OD_negative control_) × 100

### 4.8. Kill Kinetics

Kill kinetics experiment was carried out at three concentrations viz. 0.5× MIC, 1 × MIC, and 2 × MIC. Test cultures at mid logarithmic phase (6 log cfu/mL) were incubated with aqueous extract at three concentrations determined by broth microdilution assay. Samples were taken out at different time intervals, serially diluted, and plated to count the colony-forming units (CFU). The bactericidal effect was determined by 99.9% reduction of bacteria (decrease > 3 Log10 of CFU/mL) compared to the initial inoculum concentration.

### 4.9. Haemolysis Assay

Ten milliliters of venous blood was drawn from the human donor directly into the K2-EDTA-coated Vacutainer tubes. Blood was centrifuged at 500× *g* for 5 min and levels of hematocrit and plasma were marked on the tube. Plasma was aspirated out and discarded into biohazardous waste. The hematocrit tube was filled with 150 mM NaCl solution up to the marked level of plasma. The tube was gently inverted a few times to ensure proper mixing and then centrifuged 500× *g* for 5 min. The washing step was repeated and the supernatant was replaced with PBS (pH 7.4). One milliliter of erythrocytes was added into 49 mL of PBS to obtain a 1:50 dilution. A hemolysis experiment was performed in a 96-well plate. Buffalo urinary aqueous extract was diluted in 190 µL of erythrocytes to obtain final test concentrations 4000, 2000, 1000, 500, 250, and 125 µg/mL. Ten microliters of 20% Triton X-100 was added in positive control wells and 10 µL of PBS was added into negative control wells. For each peptide and control, samples were loaded in triplicate. Using a multichannel pipette, 190 µL of homogenous erythrocytes was added to each well. The plate was incubated at 37 °C for one hour on an orbital shaker. The plate was centrifuged at 500× *g* for 5 min to pellet non-lysed erythrocytes. One-hundred microliters of supernatant was transferred into clear flat bottom 96-well plate. The absorbance of the supernatant was measured by the Tecan nanoquant 96-well plate reader at 541 nm. Percent hemolysis was calculated using following formula:

% haemolysis = (OD_Treated_ − OD_diluent_)/(OD_TritonX100_ − OD_Blank_) × 100

### 4.10. Cytotoxicity Assay

BuMEC cells were trypsinized and seeded in a 96-well plate at a concentration of 1 × 10^4^ cells/well in a 100 μL culture medium. The plates were incubated for 24 h at 37 °C under 5% CO_2_ to obtain an even number of cells in all the wells. After 24 h of incubation, the old medium was removed from each well carefully without disturbing the monolayer. A dilution series of cow urinary peptides were prepared in a different 96-well plate to obtain the final test concentrations of 5000, 2500, 1250, 625, 312.5, and 156.25 µg/mL. Ten percent DMSO was dispensed into positive control wells, and 100 μL of culture medium was added to negative control wells. The experiment was performed in triplicate. The urinary peptide dilution was then dispensed into designated wells in a 96-well culture plate and incubated for another 24 h at 37 °C under 5% CO_2_. After 24 h incubation, a culture medium containing a test concentration of urinary peptides was replaced with fresh culture medium. Twenty microliters of MTT reagent (5 mg/mL) was added and incubated for 3 h at 37 °C in the CO_2_ incubator. The MTT solution was removed carefully without disturbing the formazan crystal. Additionally, 150 μL of DMSO was added to each well and the plate was agitated on an orbital shaker for 15 min to solubilize the formazan crystals. The absorbance was measured by a Tecan nanoquant 96-well plate reader at a wavelength of 565 nm, with a reference wavelength of 620 nm. Percent BuMEC inhibition was calculated using following formula:

% BuMEC survival = (OD_Treated_ − OD_Blank_)/(OD_Untreated_ − OD_Blank_) × 100

% BuMEC inhibition = 100-% BuMEC survival

## 5. Conclusions

Our study demonstrated that the urinary aqueous extract of an animal contains small molecular weight endogenous peptides. Antimicrobial assays showed that a urinary peptides extract is responsible for the antimicrobial activity, and further MS/MS analysis resulted in the identification of thousands of peptide sequences. The peptide sequences originating from different proteins of local and systemic origins find their way into urine once they are released by the proteases from their precursors. These peptide sequences were generated by the action of 76 proteases predicted in our data. Proteases provide a deep insight into animal pathophysiology and their differential expression can be associated with certain conditions. In our study, we found proteases that might be involved in the generation or activation of antimicrobial peptides or other arms of innate immunity. The peptide sequences possess different bioactivities on the basis of their amino acid composition. Our finding in buffalo is very similar to our finding in cows. Both species hail from the Bovidae family and the similarity between these two groups in different aspects of studies could be due to the fact that these two species are estimated to have had a common ancestor 5 million years ago. Summarily, the trend in the amino acid composition, physicochemical parameters, and trends in protease usage can be explained by the close relatedness of the two species.

## Data Availability

Data is unavailable due to privacy.

## Supplementary Materials

The following supporting information can be downloaded at: https://www.mdpi.com/article/10.3390/antibiotics13040299/s1, Table S1: List of the Murrah buffalo urinary peptides; Table S2: List of the Murrah buffalo urinary proteins; Table S3: List of bioactive peptide sequences; Table S4: List of Proteases identified by Proteasix responsible for the release of urinary peptides from the parent proteins.
